# The total solar irradiance during the recent solar minimum period measured by SOHO/VIRGO

**DOI:** 10.1038/s41598-021-87108-y

**Published:** 2021-04-09

**Authors:** W. Finsterle, J. P. Montillet, W. Schmutz, R. Šikonja, L. Kolar, L. Treven

**Affiliations:** 1 Physikalisch-Meteorologisches Observatorium Davos/World Radiation Center (PMOD/WRC), Davos, Switzerland; 2 Department of Computer Science, Eidgenössische Technische Hochschule (ETH), Zurich, Switzerland

**Keywords:** Space physics, Astronomical instrumentation, Solar physics

## Abstract

Various space missions have measured the total solar irradiance (TSI) since 1978. Among them the experiments *Precision Monitoring of Solar Variability (PREMOS)* on the PICARD satellite (2010–2014) and the *Variability of Irradiance and Gravity Oscillations (VIRGO)* on the mission *Solar and Heliospheric Observatory*, which started in 1996 and is still operational. Like most TSI experiments, they employ a dual-channel approach with different exposure rates to track and correct the inevitable degradation of their radiometers. Until now, the process of degradation correction has been mostly a *manual* process based on assumed knowledge of the sensor hardware. Here we present a new *data-driven* process to assess and correct instrument degradation using a machine-learning and data fusion algorithm, that does not require deep knowledge of the sensor hardware. We apply the algorithm to the TSI records of PREMOS and VIRGO and compare the results to the previously published results. The data fusion part of the algorithm can also be used to combine data from different instruments and missions into a composite time series. Based on the fusion of the degradation-corrected VIRGO/PMO6 and VIRGO/DIARAD time series, we find no significant change (i.e $-0.17\pm 0.29$ W/m$^2$) between the TSI levels during the two most recent solar minima in 2008/09 and 2019/20. The new algorithm can be applied to any TSI experiment that employs a multi-channel philosophy for degradation tracking. It does not require deep technical knowledge of the individual radiometers.

## Introduction

Machine learning and data fusion are two major components of Big Data to analyse vast amount of data and to look for trends or patterns, which have found many applications especially in the industry, opening new niche markets attracting more consumers^1^. In geosciences and solar sciences, the main goal is to process large data sets collected by satellites and/or other networks of receivers^2^. Those various data sources can hide common patterns, long-term trends or abnormal behaviors which can be detected using machine learning and data fusion algorithms. From a data science perspective, these algorithms allow the extraction of relevant information from various data sets through the adoption of intelligent monitoring (machine learning) based on a probabilistic analysis of the data sets. This data-driven approach reduces the “human factor” which can introduce biases in the data analysis.

### Total solar irradiance data sets

Total solar irradiance (TSI) measurements are important for both solar physics and climate sciences. In the Earth radiation budget, the TSI affects directly the relative significance of natural and anthropogenic contributions to the climate change^3^. To date, the longest TSI time series have been produced by the ACRIM (JPL)^4^, VIRGO (PMOD/WRC together with the Institute Royal de Méteéorologie Belgique, IRMB)^5–7^, and the Laboratory for Atmospheric and Space Physics (LASP, University of Colorado)^8,9^ teams. The ACRIM team provides the data records collected by the Solar Maximum Mission SSM/ACRIM1 (1980–1989), the Upper Atmosphere Research Satellite UARS/ACRIM2 (1991–2001), and the ACRIMSAT/ACRIM3 (1999–2013) missions. The VIRGO team produces the TSI record from the DIARAD (IRMB) and PMO6 (PMOD/WRC) radiometers on board of the SOHO/VIRGO experiment (1996–present), the PICARD/PREMOS (2010–2014), and the NORSAT1/CLARA (2017–present)^10^. LASP provides the TSI time series from the SORCE/TIM instrument (2003–2020), TCTE/TIM (2013–2019) and TSIS-1/TIM (2018 to present).

### Instrument degradation and TSI composites

Degradation of radiometers on board of satellites due to UV/EUV radiation has been a subject of research and several methods for its correction have been proposed^5,11^. Each of the data sets listed above consist of the TSI measurements from an *active* (continuously operated) and at least one *back-up* (occasionally operated) channel. The instrument degradation is then assessed by comparing the measurements of the active channel to the occasionally operated back-up channel(s). The exact procedure is a matter of personal judgement by the instrument team and is often evolving over the lifetime of each experiment. Correction of degradation is particularly important when comparing and/or combining the TSI measurements from different missions into a single *composite* time series. During the last 3 decades, different groups have produced individual TSI composites from different TSI data sets, based on different assumptions and often using personal judgement when processing the data sets^4,5,12^. The scientific community is debating the different solutions with respect to which of the composites should be used as reference. More recently, Ref.^13^ published a new method to combine the data from different sensors in an objective way using a maximum likelihood estimator. However, this new method still relies on the time series produced by the individual instrument teams, which often suffer from at least some level of subjectivity in the assessment and correction of instrument degradation.

Here, we present an algorithm based on machine learning and data fusion to process the TSI observations without filtering or applying any sort of data pre-processing which can be assimilated to “human refinement”. Based on the PREMOS/PMO6 data set, we demonstrate the robustness of our approach to correct for sensor degradation in TSI radiometers. We then apply the algorithm to the VIRGO/DIARAD and VIRGO/PMO6 data sets and combine them into a new *VIRGO TSI composite*. The VIRGO TSI composite has been the major contributor to the widely used *PMOD composite*, which was never updated since the demise of its maintainer and former PI of VIRGO, Dr. Claus Fröhlich (1936–2019).

### Data fusion

Various data fusion techniques are already available in many engineering fields^14^. Our algorithm to merge (fuse) the simultaneous measurements from two sensors is based on maximum likelihood and Gaussian processes in order to model the stochastic noise intrinsic to the TSI observations (e.g., Gaussian noise). The underlying (Bayesian) probabilistic framework guarantees the robustness of our approach^15^. We use data fusion to merge the active and back-up channels into the degradation-corrected time series of the respective TSI experiment. Ultimately, the data fusion algorithm can also combine the simultaneous observations from *different* TSI experiments in order to produce a composite based on the stochastic noise properties relative to each instrument. We demonstrate the concept by fusing the VIRGO/PMO6 and VIRGO/DIARAD time series.

## Observations and methodology

### The TSI observations

The TSI has been recorded by several space missions since the late 1970s. The time series from the various instruments are almost contiguous. The VIRGO experiment on the ESA/NASA SOHO Mission was launched in December 1995 and started observations in January 1996. The VIRGO experiment carries two different TSI radiometers, DIARAD, which was designed and built by IRMB, and PMO6-V by PMOD/WRC. A detailed description of the instrumentation is given in Ref.^16^. The DIARAD observations are processed at IRMB^12^, while the PMO6-V observations are processed at PMOD/WRC. We also use the observations from PICARD/PREMOS, which have also been processed at PMOD/WRC. For reference we compare our results to SORCE/TIM^17^ and ACRIMSAT/ACRIM3^4^. Table 1 displays the instruments and the processing centers providing the observations used in this work. The data processing, including corrections for all a priori known influences such as distance from the sun (normalized to 1 AU), radial velocity to the sun, and thermal, optical, and electrical corrections, are usually implemented by each processing center, leading to level-1 time series. On the other hand, the degradation of the radiometer is caused by long-term sensitivity changes of the sensor and/or drifts of the electrical characteristics. The degradation is assessed a posteriori based on the relative change of the active channel with respect to the back-up channel(s). The change of sensitivity is generally related to the changes in the absorptance of the black coating of the cavity^18^, or the loss of glossiness of specular paint. Both effects are thought to be caused by the UV and EUV radiation^16^.

**Table 1:** Overview of the data sets used in this study.

Mission/experiment/instrument	Version	Start date	End date
SOHO/VIRGO (soft)	a	01/1996	10/2019
SOHO/VIRGO/PMO6	6 and 7	01/1996	05/2018 and 10/2018
PICARD/PREMOS (soft)	a	06/2010	03/2014
PICARD/PREMOS/PMO6 (v1)	1	06/2010	03/2014
SOHO/VIRGO/DIARAD (R)	a	01/1996	10/2017
SOHO/VIRGO/DIARAD (L)	a	01/1996	10/2020
ACRIMSAT/ACRIM3	11/13	04/2000	11/2013
SORCE/TIM	18	02/2003	02/2020

Note that *soft* is the data analysed with our software, *PMO6* is the TSI time series released by PMOD, the *DIARAD* data set are provided by the Royal Meteorological Institute of Belgium^12^, whereas *SORCE/TIM* time series are downloaded from The University of Colorado Boulder^17^ and *ACRIM* from http://www.acrim.com.

Note that DIARAD-R does not have any observations after 10/2017, whereas we use in this study DIARAD-L observations until 10/2020.

#### The VIRGO/PMO6-V observations

On VIRGO, two PMO6-V radiometers (i.e. PMO6-VA and PMO6-VB) of the same design and black coating (Aeroglaze Z302) are used as ’active’ and ’back-up’ instruments. The back-up instrument PMO6-VB is operated only rarely to keep its degradation low compared to PMO6-VA. PMO6-VB is operated once every ten days during 39 min. Before 6 July 1996, PMO6-VB was operated 3 times per day for 39 min. Further details can be found in Ref.^16^.

The degradation function of PMO6-VA has been previously determined and published online as versions PMO6-v6 and -v7. The assumptions vary slightly between both versions, but we recall the main three hypotheses: (a) the sensitivity decreases with exposure to solar radiation and is modelled by an exponential, (b) there is an early increase in sensitivity during the first few days modelled with an exponential, (c) a non-exposure dependent degradation of $-0.3$ ppm/mission day is found by comparing PMO6-VB with the back-up channel of VIRGO/DIARAD (DIARAD-R). It leads to correct the level-1 PMO6-VA observations with a sum of exponential and linear functions. The process is fully described in Refs.^7,16^.

#### The VIRGO/DIARAD observations

DIARAD is the second type of radiometer on the SOHO/VIRGO experiment. It features two radiometric channels (DIARAD-L and DIARAD-R) and uses a different black coating than the PMO6-V (3M Nextel VELVET Black 2010). The back-up channel DIARAD-R is operated every 60 days during 90 min (30 min until 7 August 1996). The degradation-corrected TSI time series are produced in a process similar to PMO6-V. Here, we use two degradation-corrected data products, namely the PMOD-v6 discussed in Ref.^7^ and the TSI composite released by IRMB^12,19^. The former will be referred in the following as PMOD-v6 and the latter as the IRMB/DIARAD product. According to Ref.^20^, the degradation correction of the IRMB/DIARAD product is based on an exponential, with various assumptions (e.g. exposure time, offsets).

#### The PREMOS/PMO6-P observatsions

Like VIRGO, PICARD/PREMOS is also equipped with two PMO6-type radiometers^21^. These are referred to as PMO6-PA (active) and PMO6-PB (back-up). The PICARD mission started in 2010 and ended in 2014. The degradation of the PREMOS/PMO6 radiometer was determined in Ref.^21^ and reevaluated by Ref.^22^.

### Robust TSI estimates with machine learning and data fusion

In this section, we produce degradation-corrected TSI time series using the active and back-up instruments/channels for each experiment (i.e. PMO6-VA and PMO6-VB, DIARAD-L and DIARAD-R, PMO6-PA and PMO6-PB). Our *first assumption* is that degradation is a function of *exposure time* only. The exposure time is estimated via a cumulative sum of the measurements recorded by each sensor. We are aware that an effective UV/EUV dose could be used instead of the exposure time. However, in practice, it turns out that using dose does not yield a significant difference in the degradation assessment^22^. Since the back-up instruments/channels (PMO6-VB, DIARAD-R, PMO6-PB) have lower observation rates (and hence exposure times) than the active instruments/channels (PMO6-VA, DIARAD-L, PMO6-PA), they degrade less rapidly. Our *second assumption* is that all instruments and channels start with no degradation and the *third assumption* is that the degradation curve is *monotonically decreasing*.

The proposed new algorithm is divided in 2 steps. The first step is modelling the degradation curve and corrects the level-1 observations, whereas the second step is the data fusion where the corrected measurements from the active and back-up instruments (or channels) are merged in order to produce a single TSI time series. The algorithm is published in Refs.^23,24^. In the following we give a brief summary.

#### Degradation modeling and correction

The three assumptions defined above can be formally be expressed in the following way for the degradation function *d*:

1 $$\begin{aligned} \begin{array}{l l} 1. &{} d=d(e_x)>0 \\ 2. &{} d(0)=1 \\ 3. &{} {\partial \over \partial e_x}d<0 \end{array}, \end{aligned}$$

where $e_x$ is the exposure time, and ${\partial \over \partial e_x}$ denotes the partial derivative. Note that for PMO6-type radiometers the observations indicate that ${\partial \over \partial e_x}d>0$ for $e_x \lessapprox 5\mathrm {\ days}$^22,25^. Because the algorithm cannot yet model such *negative degradation*, we still use the method described in Ref.^22^ to manually correct this so-called *early increase* of PMO6-type radiometers based on a linear fit of the active vs. backup channels over the first five days of exposure time of the active channel. A detailed description of how the early increase is corrected is given in the appendix. A future version of the algorithm will be able to model and correct for non-monotonic degradation.

If $e_a$ and $e_b$ are the respective exposure times of the active and back-up sensors, *s* the “true” TSI signal (without degradation), and $\varepsilon _a$ and $\varepsilon _b$ the measurement noise, then the actual signals *a* and *b* (with degradation) which each sensor measures at time *t* are:

2 $$\begin{aligned} \left\{ \begin{array}{l l} a(t) = s(t) d(e_a(t)) + \varepsilon _a(t), &{} \varepsilon _a(t) \sim \mathcal {N}(0, \sigma ^2_a) \\ b(t) = s(t) d(e_b(t)) + \varepsilon _b(t), &{} \varepsilon _b (t) \sim \mathcal {N}(0, \sigma ^2_b) \\ \end{array} \right. , \end{aligned}$$

with the assumption that the noise $\varepsilon$ is zero-mean Gaussian distributed (with variance $\sigma ^2$) and independent for each instrument and channel. The active and back-up radiometers are technically identical, therefore the degradation function is assumed to be identical for both channels.

Now, the goal is to estimate $d(e_x)$ from the observations *a*(*t*) and *b*(*t*). Neither the true signal *s*(*t*) (i.e. without noise and degradation) nor the degradation function $d(e_x)$ are known throughout the process. We determine *d* solely from the ratio of the signals $r(t)={a(t) / b(t)}$. To estimate $d(e_x)$ we propose an iterative process to correct the signals *a*(*t*) and *b*(*t*) as follows:

3 $$\begin{aligned} r_p(e_a(t))={a_0(t) \over b_p(t)},\quad b_{p+1}(t)={b_0(t) \over r_p(e_b(t))}\quad p=0,1,2,\ldots \end{aligned}$$

where $a_0(t)=a(t)$, $b_0(t)=b(t)$. As shown in^24^, the ratio $r_p(e_x)$ converges towards the degradation function $d(e_x)$:

4 $$\begin{aligned} \lim _{p \rightarrow \infty }r_p(e_x)=d(e_x) \end{aligned}$$

In practice, the iterative process described in Eq. (3) is performed by *fitting* of a function $d_\theta (e_a(t))$ (parametrized by $\theta$) to $r_p(e_a(t))$ by minimizing the objective function:

5 $$\begin{aligned} \min _{{\theta }}{\sum _t (d(e_a(t),{\theta })-r_p(e_a(t)))^2}\,, \end{aligned}$$

For our type of observations, we empirically established via simulations that $d_\theta$ is best described as an *isotonic* function^26^, although *monotonic* and *smooth monotonic* functions have also been tried. Once $d_\theta$ is estimated iteratively, the measurements *a*(*t*) and *b*(*t*) can be corrected using:

6 $$\begin{aligned} \left\{ \begin{array}{l l} a_c(t)={a(t) \over d_\theta (e_a(t))} \\ b_c(t)={b(t) \over d_\theta (e_b(t))} \\ \end{array}\right. \end{aligned}$$

The algorithm to extract the corrected measurements is displayed in the appendices together with the definition of the isotonic functions (monotonic and smooth monotonic).

#### Data fusion

After correcting the measurements ($a_c(t_i), b_c(t_i)$), the Eq. (2) becomes:

7 $$\begin{aligned} \left\{ \begin{array}{l l} a_c(t) = s(t) + \varepsilon _a(t), &{} \varepsilon _a (t) \sim \mathcal {N}(0, \sigma ^2_a) \\ b_c(t) = s(t) + \varepsilon _b(t), &{} \varepsilon _b (t) \sim \mathcal {N}(0, \sigma ^2_b) \\ \end{array} \right. \end{aligned}$$

The data fusion aims at merging the corrected observations from the two channels in order to get a reliable estimate of the true signal *s*, knowing that the underlying process model of *s* is random and unknown. Therefore, we formulate two assumptions: a/the solar cycle is not a deterministic signal and its variations are random (no a priori knowledge). *s* is then assumed to be a Gaussian process (GP) with zero mean and a covariance function $k_{\alpha }(.,.)$ (or kernel); b/ because we consider the noise on the measurements zero mean Gaussian distributed, then we can estimate the parameters of the model of *s*(*t*) via maximum likelihood estimator. We then formulate $\mathbf {s} \sim GP (0, k_{{\alpha }}(.,.))$. However, the main limitation of GPs is that given *n* observations, the inverse of the $n \times n$ covariance matrix must be computed. Time complexity of such operation is of the order of $O(n^3)$, which is not scalable, especially when computational resources are limited. The VIRGO/SOHO mission has been recording observations at a high rate (i.e. 1 min sampling of PMO6-VA) for a long time (since 1996), therefore we deal with large TSI data set (i.e. $n >10^7$). We therefore approximate the exact GP with a Sparse Gaussian Process^24^ (SPG) to construct a lower bound for the log-likelihood $\log {p(\mathbf {y}|\mathbf {x})}$. $\mathbf {x}$ and $\mathbf {y}$ are the concatenation of times, $\mathbf {x} = [t_i, t_i], i=1\ldots n$, and corresponding corrected observations $\mathbf {y} = [a_c(t_i), b_c(t_i)]$. The mathematical formula is displayed in the appendices.

It is important to emphasize that the training of $k_{{\alpha }}$ with the so-called “inducing points” is to learn about the stochastic properties of the data, which allows to take into account short-term correlations in the observations and a reasonable approximation of *s*. Thus, our simulations use 2500 inducing points which is a trade-off between modelling well all the processes and avoiding long computing time (i.e. no more than 10h) with a regular desktop computer (i.e. 16G RAM, 4 cores). Note that the final time series has an hourly rate in order to avoid large data set (i.e. > 100 MB).

## Results and discussion

### Degradation correction of PREMOS/PMO6 measurements

**Figure 1:**
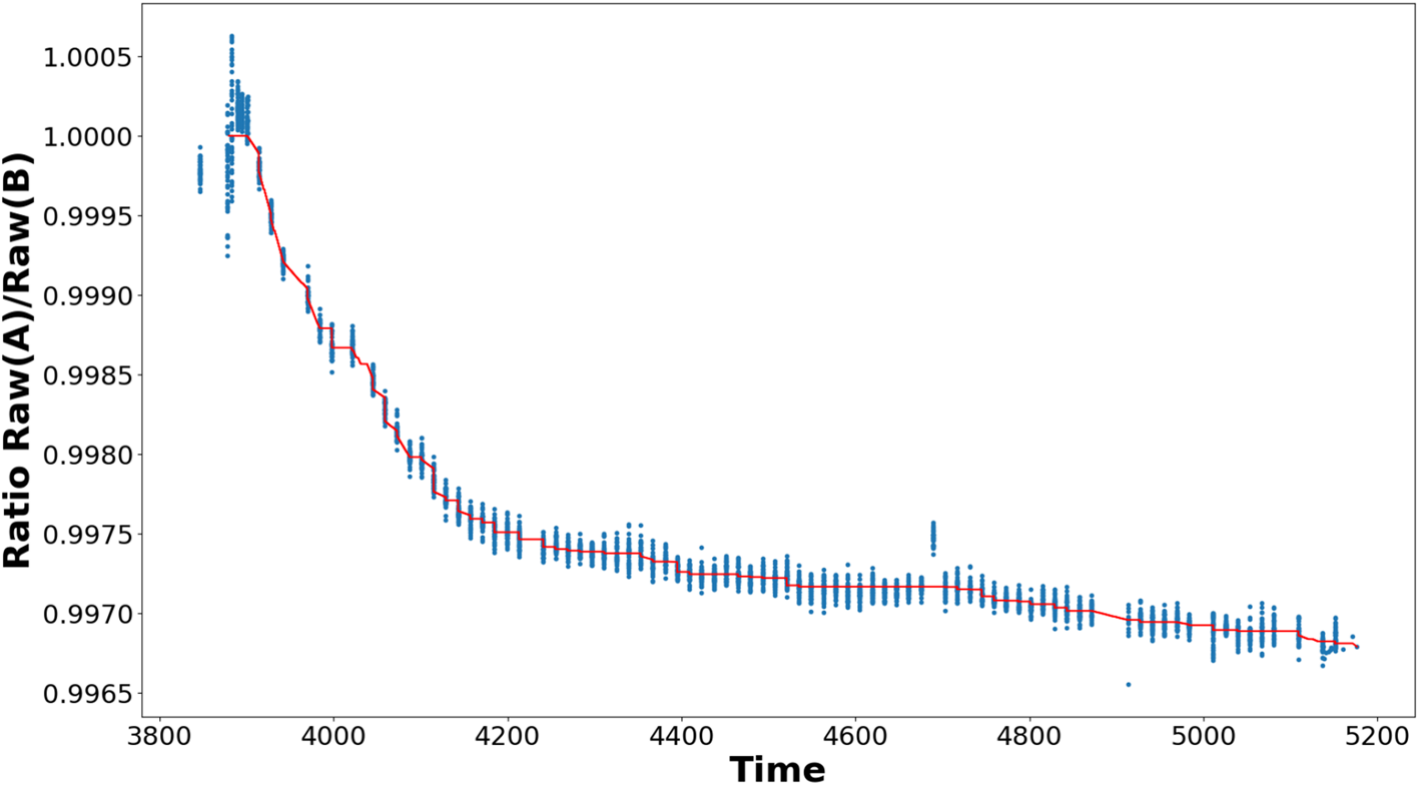
Ratio of the raw measurements PMO6-PA and PMO6-PB as a function of time (days since 1 January 2000). The red curve is the degradation correction function. Note that the early-increase was corrected manually before running the degradation correction algorithm. The degradation curve thus only starts after the early-increase phase of PMO6-PA (see text for details on the “early increase” phenomenon).

**Figure 2:**
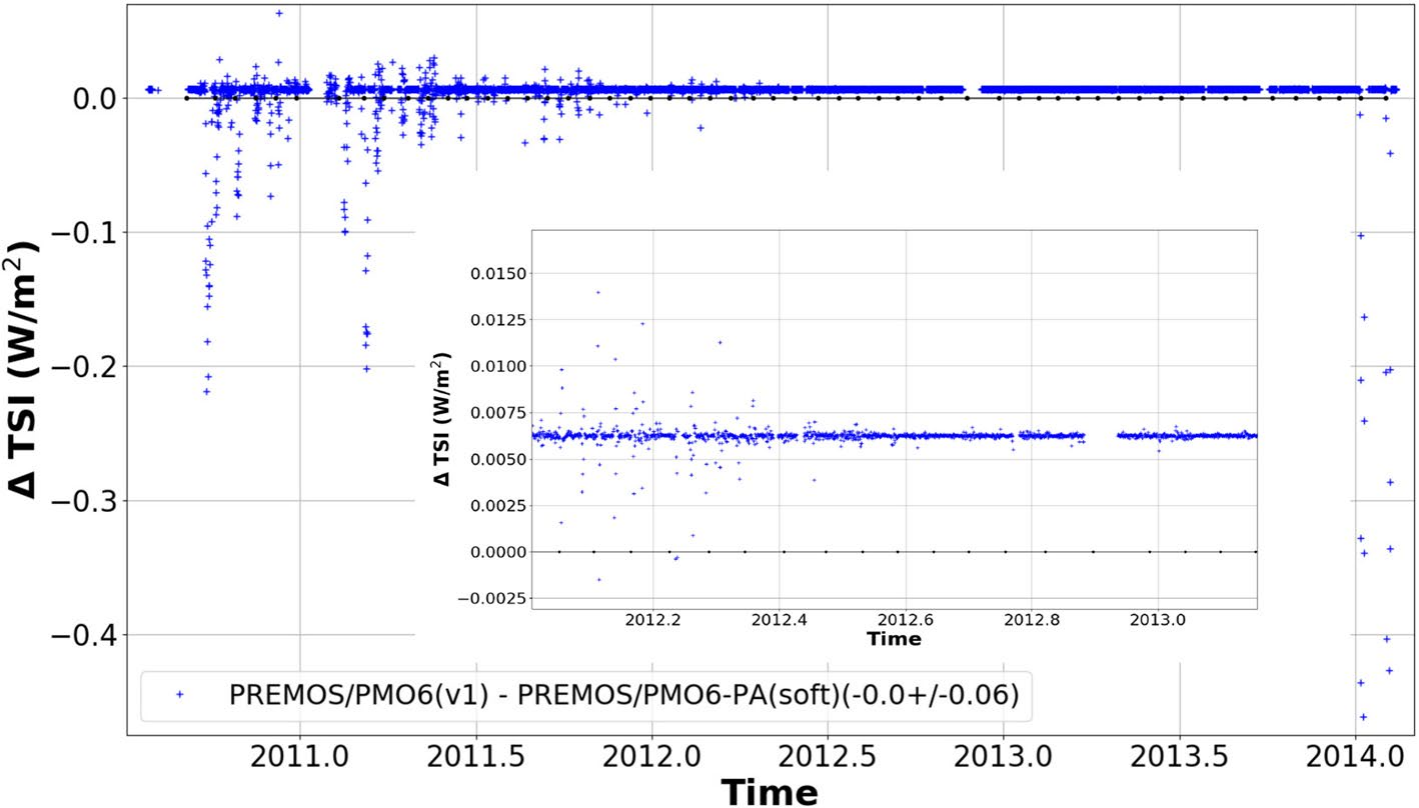
The difference between the TSI time series calculated by the machine-learning and data fusion algorithm and the manually determined solution by Ball et al.^22^ (PREMOS/PMO6(v1)). The tiny offset ($~0.0062\mathrm {Wm^{-2}}$) between both solutions is well within the approximation uncertainties for both degradation curves. The occasional outliers are caused by different smoothing in both procedures. The zoom-in (insert) gives a better impression of the relative long-term stability between both solutions. There is no appreciable trend in our new solution compared to the original PREMOS-v1.

In the previous sections, we have introduced the algorithm to estimate the degradation function based on the ratio of the raw measurements from the active and back-up channel. We apply this algorithm to the PREMOS PMO6-PA and PMO6-PB level-1 data after manually correcting the early increase (see appendix). The Fig. 1 displays the ratio of level-1 observations of PMO6-PA and PMO6-PB as a function of time together with the degradation function determined by our algorithm using *isotonic* regression (red curve). To assess the validity of our new degradation function, we compare it to the previously published solution. We find that our algorithm reproduces the well-established degradation curve (at the level of 0.0062 W/m$^2$) determined for PREMOS/PMO6 in a classical approach^22^ with no appreciable relative trend between both solutions (see Fig. 2). Over the full PREMOS mission the new TSI time series agrees with previous release in absolute value $\sim 0.12$ W/m$^2$ RMSE (PREMOS-v1, see Figure A.5).

### Degradation correction of VIRGO measurements

**Figure 3:**
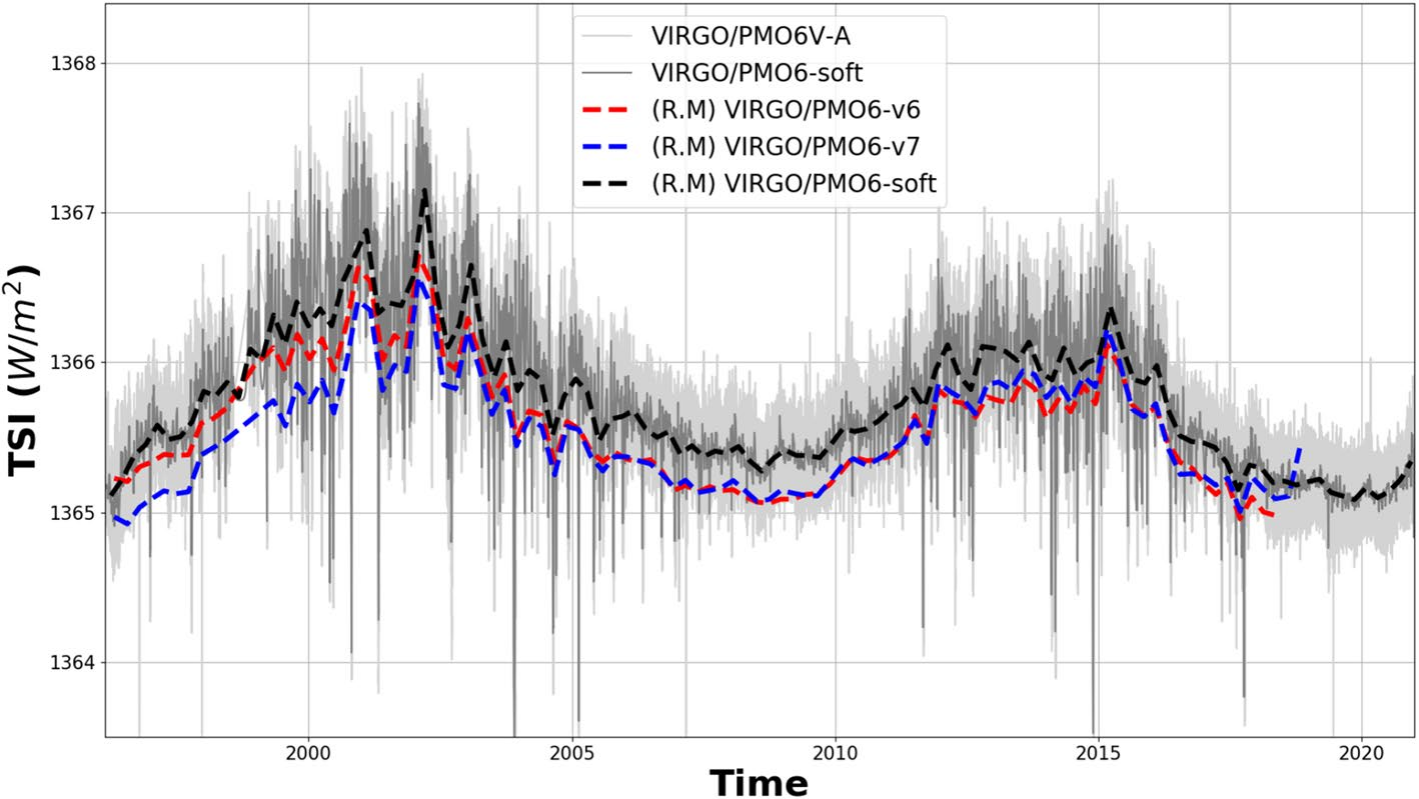
The degradation-corrected PMO6-VA time series (light grey), the fused PMO6-VA and -VB (PMO6-soft, dark grey) and the previous versions of the VIRGO/PMO6 degradation corrected TSI time series (PMO6-v6 (red), PMO6-v7 (blue)). The dashed lines are 81-day running means (R.M.).

**Figure 4:**
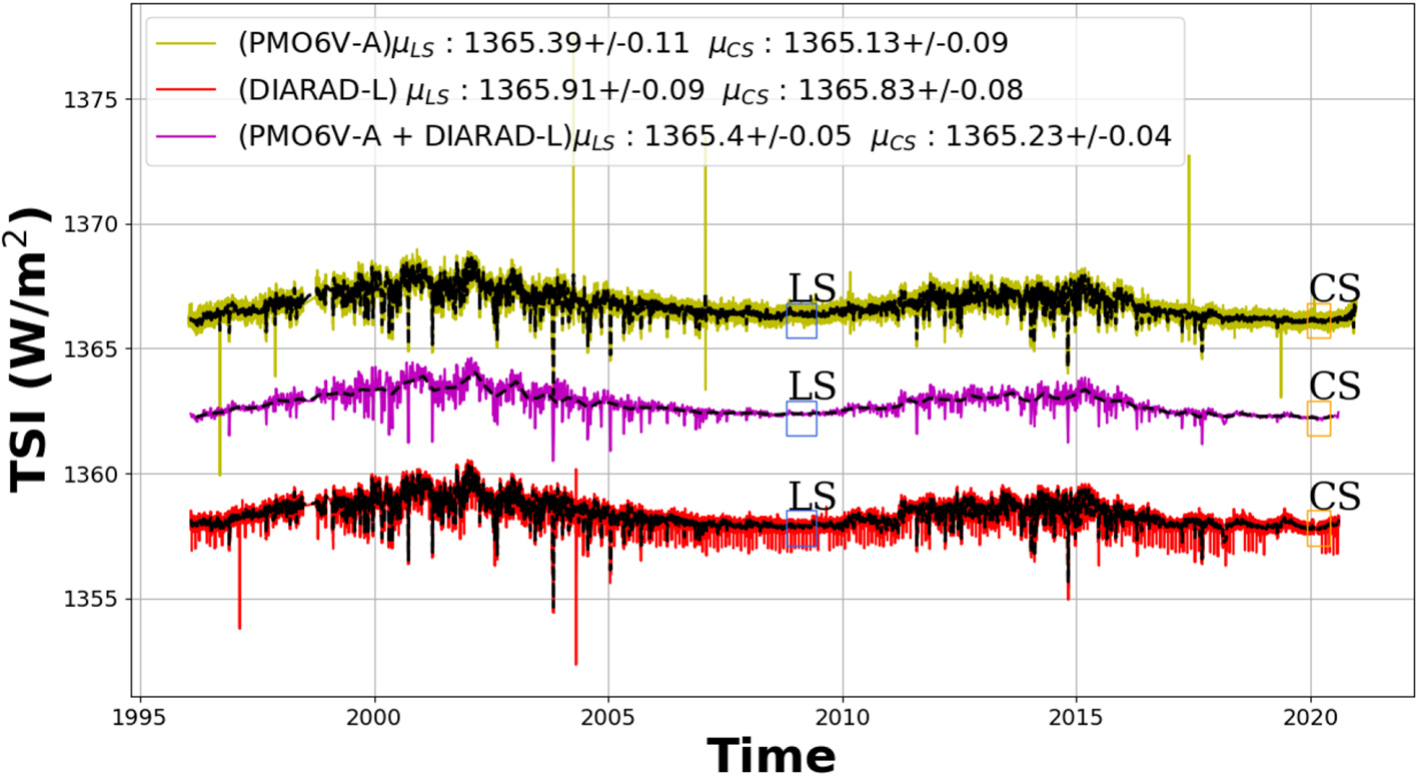
The new VIRGO TSI composite (purple) based on the fusion of the degradation-corrected DIARAD-L (red) and PMO6-VA (green). In the case of DIARAD, an extrapolated degradation curve was used to correct DIARAD-L after the failure of the backup channel (DIARAD-R) in 2017. Note that the last solar minimum (LS—blue box) and the current one (CS—orange box) are estimated for both 2008–2009 and 2019–2020. The time series are offset for clarity. In the inset we give the mean TSI values for each time series ($\mu$).

The VIRGO experiment provides the longest TSI time series to date, covering more than two 11-year solar cycles. Comparing the TSI levels during consecutive solar minima is thought to provide important information on secular changes in the radiative output of the Sun. It is crucial to accurately model and correct the degradation of the VIRGO radiometers before we can assess the variability of solar minimum levels of the TSI. Previously, the degradation of the VIRGO time series was corrected in a highly sophisticated, mostly manual procedure which has become increasingly complex as the time series grew longer and additional instrumental effects had to be considered in order to accurately model the degradation curve^5,7,11^. We now use our machine-learning algorithm to solve the degradation issue in an automated, fully reproducible process for both VIRGO/PMO6 and VIRGO/DIARAD time series. Manual correction of the early increase was applied to the VIRGO/PMO6 time series as explained in the previous section. The degradation-corrected TSI time series of VIRGO/PMO6 is shown in Fig. 3 together with the previous solutions PMO6-v6 and -v7. The degradation-corrected DIARAD-L time series is shown in Fig. 4.

Table 2 shows how our solutions compare with previous data releases in terms of the average TSI levels during the solar minimum in 2008/2009, at the transitions from solar cycle 23 to 24. The solar minimum period (2008 September 20–2009 May 5) was chosen according to the ISSI team meetings 2012 and 2013^8,9,27^.

**Table 2:** Comparison of the TSI levels (mean $\mu$ and standard deviation $\sigma$) during the two most recent solar minimum periods between solar cycles 23/24 and 24/25, respectively, as indicated by “LS” and “CS” in Fig. 4.

Solar minimum TSI level ($\mu \pm \sigma$ [W/m$^2$])		Solar cycle			
		23/24		24/25	
		Classic	Machine-learning	Classic	Machine-learning
**TSI experiment**					
SORCE/TIM		$1360.53 \pm 0.04$	–	$(1360.66\pm 0.04)$	–
ACRIM3		$1360.78 \pm 0.06$	–	–	–
VIRGO/PMO6-v6		$1360.59 \pm 0.07$	$1360.79 \pm 0.06$	–	$1360.53 \pm 0.04$
VIRGO/PMO6-v7		$1360.63 \pm 0.07$		–	
VIRGO/DIARAD		$1361.39 \pm 0.04$	$1361.31 \pm 0.09$	–	$\mathbf1361.23 \pm 0.08$
IRMB/DIARAD		$1361.26 \pm 0.05$	–	–	–
VIRGO composite		$1360.41 \pm 0.05$	$1360.80 \pm 0.05$	–	$1360.63 \pm 0.04$
				–	$(1360.65 \pm 0.04 )$

We show the results from various TSI time series produced by PMOD/WRC and other groups (LASP, ACRIM, IRMB) from various missions and experiments.

The dash (–) means no estimate is available, “classical” and “machine-learning” refer to the method that was used for correcting the degradation.

Note that for easier comparison all TSI values from the VIRGO experiment have been scaled to the “new VIRGO” scale^25^, which means the machine-learning results are offset by $-4.6$ W/m$^2$ compared to those stated in Fig. 4.

The SORCE/TIM time series used in this work ends in February 2020.

The cycle 24/25 minimum value for SORCE/TIM is thus not averaged over the same period as for VIRGO.

All VIRGO values would be 0.2 W/$m^2$ *higher* if evaluated over the same period as SORCE/TIM.

Values averaged over the shorter time period are put in brackets ().

In Table 2 all VIRGO data are expressed according to the “new VIRGO” scale^25^ to allow easier comparison with the data sets of SORCE/TIM and ACRIM3. The scale offset is explained by different reference scales of both data sets. SORCE/TIM is traceable to SI while SOHO/VIRGO was calibrated against the World Radiometric Reference (WRR), which is offset by $0.34\%$ with respect to SI^28^, resulting in an offset of $-4.6$ W/m$^2$. During solar minimum 23/24 the previous (“classical”) PMO6-v6 and -v7 releases and the new (“machine-learning”) solution for VIRGO/PMO6 differ on the order of magnitude of $\sim 0.2$ W/m$^2$, with the “classical” TSI values being lower. This difference is just within the 1-sigma interval of 0.2 W/m$^2$, which we define as the inter quantile range (i.e. difference between the 25th and 75th percentile, see Figure A.5).

For DIARAD the machine-learning algorithm suggest a TSI level for solar minimum 23/24 which is between the “classical” solutions provided by IRMB (0.05 W/m$^2$ *lower*) and PMOD/WRC (0.08 W/m$^2$ *higher*).

Over the full VIRGO mission, we have found that the new TSI time series’ agree with previous releases by PMOD/WRC and IRMB in absolute value between $\sim 0.1$ W/m$^2$ RMSE (VIRGO/DIARAD) and $\sim 0.25$ W/m$^2$ RMSE (PMOD-v7, Figure A.5).

### Data fusion and new TSI composite

We use the data fusion process not only to merge the degradation-corrected time series of the active and back-up channels into a single time series for each TSI experiment, but also to produce the new VIRGO TSI composite from DIARAD and PMO6-V. More generally, the fusion process can be applied to any two *simultaneous* time series from different TSI experiments with different noise variances to produce a TSI composite. Unfortunately, the DIARAD-R sensor does not record any observations since early 2018 due to a technical issue. Since then, we can no longer merge the DIARAD time series from fusing DIARAD-L and DIARAD-R. Therefore, we produce the new VIRGO TSI composie from fusing only the degradation-corrected PMO6-VA and DIARAD-L time series instead. To produce the degradation-corrected DIARAD-L, we first estimate the degradation function using both sensors (DIARAD-L, DIARAD-R) for the time when they are available. We then extrapolate with a third order polynomial the degradation function in order to correct the DIARAD-L time series in its full length.

Before the fusion, we align the VIRGO/DIARAD composite at the same nominal TSI value as VIRGO/PMO6 corresponding to the last solar minimum (i.e. 1365.39 W/m$^2$). We can then produce the VIRGO TSI composite by fusing the degradation-corrected PMO6-VA and DIARAD-L observations (Fig. 4). The mean value and the standard deviation of this *new VIRGO TSI composite* have similar characteristics than the ones estimated for VIRGO/DIARAD and VIRGO/PMO6.

The difference in the TSI levels during the last two solar minima are $-0.26$ W/m$^2$ (PMO6-VA) and $-0.08$ W/m$^2$ (DIARAD-L), respectively (empirical uncertainties based on combined standard deviations of both time series during the solar minimum periods). For the cycle 24/25 solar minimum we chose the period from 2019 Nov 1 to 2020 May 1, during which virtually no signs of solar activity appear in neither the Solar Sunspot Number nor the TSI measurements from VIRGO/PMO6. We estimate the empirical cycle-to-cycle stability of our new degradation algorithm by comparing the VIRGO TSI composite to the independent data set from SORCE/TIM. In 2008/09 VIRGO reads 0.27 W/m$^2$ *higher* than SORCE/TIM, in 2019/20 the VIRGO reads 0.01 W/m$^2$ *lower* than SORCE/TIM, resulting in a relative trend of $-0.28$ W/m$^2$ of VIRGO with respect to SORCE/TIM (see Table 2). We take this trend as the empirical uncertainty of the long-term stability of the machine-learning and data fusion algorithm. Together with the empirical standard deviations of both time series (i.e. 0.04 W/m$^2$ for SORCE/TIM and 0.05 W/m$^2$ for the VIRGO composite, see Table 2) the resulting uncertainty is $\sqrt{0.28^2+0.04^2+0.05^2)}=0.29$. From the VIRGO TSI composite, we thus find a non-significant minimum-to-minimum variation between 2008/2009 and 2019/2020 of $-0.17 \pm 0.29$ W/m$^2$.

Note that Figure A.4 in the appendices show the comparison between all the data sets and our new TSI time series.

**Figure 5:**
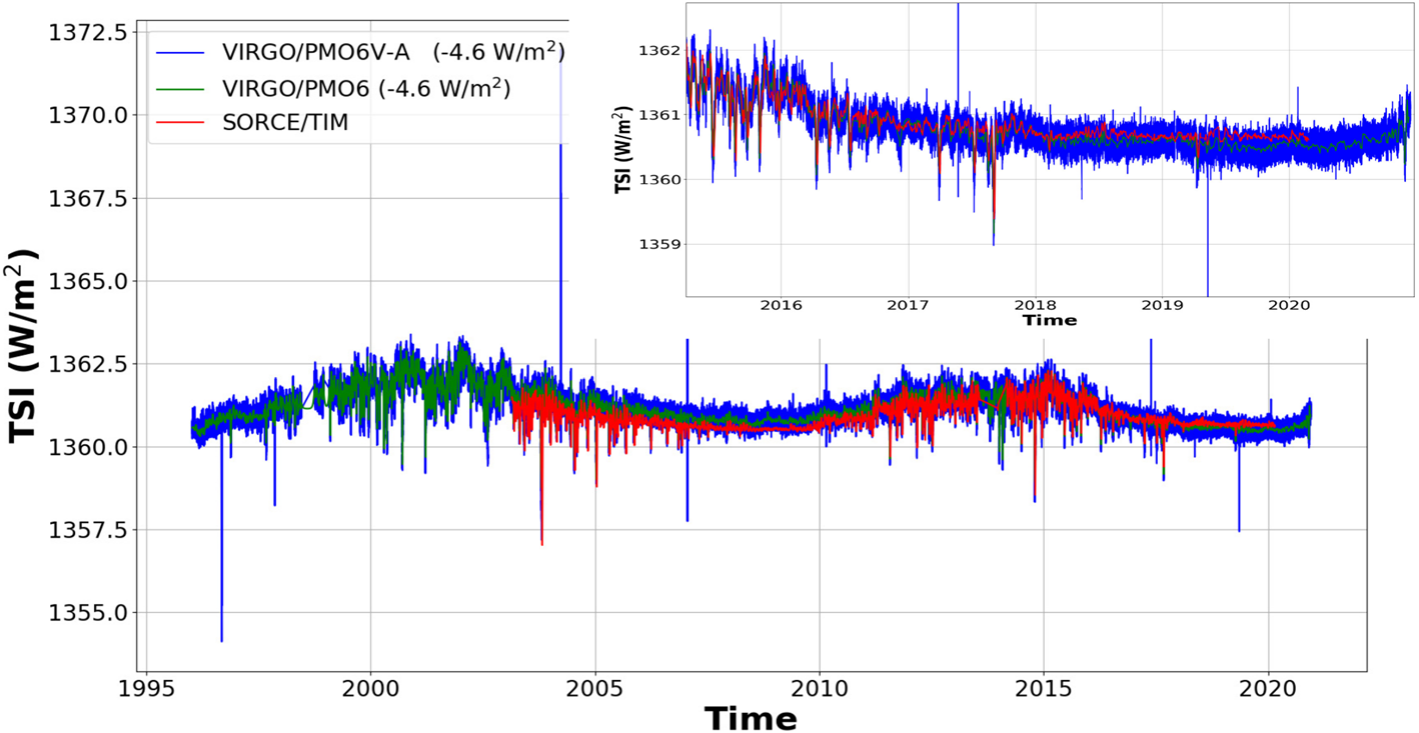
The degradation corrected PMO6-VA (blue) and the PMO6-V composite (PMO6-VA + PMO6-VB, green). For comparison we also show the SORCE/TIM time series. The inset zooms in on the period of the last solar minimum.

We note that the agreement with TIM has improved compared to previous version PMO6-v7 (Fig. 5). This is however fully attributable to an improved temperature correction algorithm which we implemented in the upstream data processing pipeline for PMO6-V. The updated temperature correction algorithm removes the slight sensitivity to the absolute temperature, which the original, purely empirical, algorithm was suffering from. The overall temperature of the VIRGO package had risen by several degrees in the course of the mission, causing a slight drift of the PM06-V measurement. This drift has now been eliminated by switching to a correction algorithm which is based solely on the temporal derivative of the heat sink temperature to correct for measurement bias. This bias is caused by slight mismatch of the thermal time constants of the measuring and compensating cavities in each PMO6-V channel. This is an a-priori effect, hence not part of the degradation correction. The concept of this new algorithm was originally developed for the PREMOS/PMO6 radiometers^27^, but never applied to the VIRGO/PMO6.

## Conclusions

The classical approach for correcting TSI instrument degradation suffers from two major weaknesses. 1/ It is subject to personal judgement, and 2/ it is based on assumed physical and photo-chemically induced changes in the sensor hardware which cannot be verified. In this study, we propose a data-driven approach of processing TSI data using machine-learning and data fusion where a small number of objective (i.e. not specific to the instrument) assumptions are sufficient to correct for instrument degradation and to produce robust TSI estimates. The first assumption is that the degradation function depends only on exposure time. Secondly, at the first epoch we have two identical, non-degraded instruments (or channels). Thirdly, the degradation is assumed to be a decreasing function. This approach largely eliminates the “human factor” and by virtue of its data-driven nature it is detached from the actual hardware changes.

From the low RMSE ($\sim 0.12$ W/m$^2$) between PREMOS-v1 and the machine-learning solution for PREMOS/PMO6 together with the absence of any appreciable long-term trend between both solutions, we conclude that the machine-learning and data fusion algorithm is capable of reproducing the degradation function with similar accuracy and precision than classical approaches. The PREMOS-v1 solution by Ball et al.^22^ is the best documented (and arguably the most sophisticated) of the four classical solutions (incl. PMO6-v6, -v7, IRMB/DIARAD) which we considered in this work. From the excellent agreement of our solution with PREMOS-v1 we conclude that for the latter three, applying the machine-learning and data fusion algorithm likely constitutes an improvement over their respective classical solutions.

We composed a new VIRGO TSI composite by fusing the degradation-corrected time series of PMO6-VA and DIARAD. The data fusion process requires co-aligning the absolute values of both time series, therefore the absolute value of the new VIRGO TSI composite is still somewhat arbitrarily chosen to match with PMO6-VA. Nevertheless, we can use the new VIRGO TSI composite to estimate drifts in TSI level between consecutive solar minima. We found no significant change ($-0.17\pm 0.29$ W/m$^2$) between the two most recent solar minimum periods (2019/2020 solar minimum vs. 2008/2009 solar minimum).

The data fusion part of the algorithm can also be used to fuse contemporaneous TSI time series from different instruments in order to produce composite time series. Future work will focus on refining the underlying assumptions of the machine-learning algorithm, including additional TSI experiments in order to feed them into the “community composite” approach by Ref.^13^, and to validate the result by comparing it to a composite based on our data fusion approach.

## Supplementary Information

Supplementary Information.

## Data Availability

The VIRGO PMO6 and VIRGO DIARAD data are accessible on the PMOD website (ftp://ftp.pmodwrc.ch/pub/data/irradiance/virgo/TSI/), whereas the IRMB DIARAD data are available on request to Dr. S. Dewitte. The data related to the monthly mean sunspot numbers are retrieved from http://www.sidc.be/silso/datafiles. The PREMOS/PICARD data can be accessed at http://idoc-picard.ias.u-psud.fr/sitools/client-user/Picard/project-index.html (It is recommended to use *Data Access* at the top left $\rightarrow$ *Datasets Explorer* and download *TSI_N2_complete*). The ACRIM3 data is downloadable at http://www.acrim.com and the latest SORCE/TIM dataset is available at https://lasp.colorado.edu/home/sorce/data/tsi-data. We downloaded the ACRIM data from http://www.acrim.com. But the site does indeed seem to no longer exist! Alternatively, https://www.ftp://ftp.ngdc.noaa.gov/STP/SOLAR_DATA/SOLAR_IRRADIANCE/ACRIM3/ could be used.
