# Supplementary material for: The total solar irradiance during the recent solar minimum period measured by SOHO/VIRGO
Source: Sci Rep. 2021 Apr 9;11:7835. doi: 10.1038/s41598-021-87108-y (PMC8035225; doi:10.1038/s41598-021-87108-y)
Supplement: Supplementary file 1 — Supplementary Information. [file 41598_2021_87108_MOESM1_ESM.pdf]

The total solar irradiance during the recent solar minimum period measured by SOHO/VIRGO

W. Finsterle[1,*], J.P. Montillet[1,*], W. Schmutz[1], R. Sikonja[2], L. Kolar[2], and L. Treven[2]

[1]Physikalisch-Meteorologisches Observatorium Davos / World Radiation Center (PMOD/WRC), Davos, Switzerland
[2]Dpt. of Computer Science, Eidgenössische Technische Hochschule (ETH), Zürich, Switzerland
[*]wolfgang.finsterle@pmodwrc.ch, jean-philippe.montillet@pmodwrc.ch

ABSTRACT

Supplementary Material/Appendices

The PICARD/PREMOS data

The PICARD/PREMOS experiment comprised twelve filter radiometer channels and a pair of TSI radiometers of the PMO6 type (PMO6-PA and PMO6-PB). The data of the filter radiometer have been published in Ref.[1]. The TSI radiometers have been calibrated in the laboratory and in front of the Sun[2]. In addition, one of the TSI radiometers, instrument PMO6-PA, has been calibrated at the TRF facility in Boulder, Colorado[3] and

was the first TSI radiometer operated in space to have been calibrated end-to-end against an SI-traceable irradiance reference prior to launch. The first light PREMOS measurements thus helped to resolve the disagreement in absolute value between the TSI measurements by SORCE/TIM[4] and other experiments, such as VIRGO, confirming the lower reading by SORCE/TIM.

The solar constant value of 1361 W/m^2 was later adopted by the IAU 2015 Resolution B3[5].

The raw telemetry data are termed level-0 data. Level-1 data are transformed to physical units and corrected for for a priori known effects. Level-2 data are corrected for the sensitivity change of the radiometers in space when exposed to sunlight. This is accomplished using ratios of two virtualy identical radiometers within the experiment, namely the "active" PMO6-PA and the "back-up" PMO6-PB radiometer. The assumption is that the radiometers are changing their sensitivities identically as a function of solar exposure. The radiometers used for PREMOS are of the same type as those used for SOHO/VIRGO[6]. This type of radiometer has been found to experience in space first a rapid sensitivity increase of ~ 0.05% and then turn over for a decline of sensitivity after a few days of exposure[7]. This behaviour is referred to as "early increase". The sensitivity change of the active PMO6-PA instrument is measured relative to the back-up instrument PMO6-PB by bi-weekly 90 min exposures, operating both instruments simultaneously, such that ratios between these instruments can be derived independently from the solar irradiance level (see Figure 2 in Ref.[8]). The sensitivity change of PMO6-PB is estimated from the first two ratios yielding a linear sensitivity increase of PMO6-PB as a function of exposure time and the corrected measurements of instrument B give a relative time series of the TSI over the mission time. In the classical approach, the measured instrument ratio over the mission time is then applied to bring instrument A on a level-2 corrected time series of absolute values of total solar irradiance. The absolute calibration is given by the first light exposure. Version 1 of PREMOS level-2 data have been published by Ref.[8].

As the switch off of the PICARD satellite was planed and known, the roles of active and back-up channels were swapped shortly before the end of the mission in order to allow the early increase of the B instrument to saturate. This burst of exposure of the B instrument allowed to investigate if the two cavities degraded similarly as a function of exposure time, in particular, if the early increase would also saturate in PMO6-PB after a few days of exposure. This assessment was published in Ref.[9] and the authors concluded that "The consistent behaviour of PREMOS-A and PREMOS-B, with respect to the other instruments, provides high confidence that PREMOS-B has degraded in the same way as PREMOS-A." The PREMOS TSI data version 1 was corrected adopting a linear sensitivity dependence of the back-up instrument B as a function of exposure time, namely an increase of 81.7 ppm per exposure day, derived from the first two irradiance rations of instrument A to B. The comparison of instrument A to SORCE/TIM during its first week of exposure yields a somewhat steeper relation, which gives about a 60 ppm larger correction at the end of the 4-year long measuring period of PREMOS. Avoiding a discussion which correction might be more reliable we simply emphasise that this difference gives a good estimate of the systematically time-dependent increasing uncertainty of the PREMOS TSI time series, which we set at 15 ppm per year. Note that the automated degradation correction algorithm discussed in this study is applied to the level-1 data, but with preliminary removing a linear sensitivity (using a third order polynomial fitting) on the back-up channel discussed in Ref.[8].

Algorithm to Correct the degraded Measurements

The Smooth Monotonic and Isotonic Functions

Monotonic or isotonic regression requires predictor values to be in a strictly increasing order $x_1 < x_2 < ... < x_n$. Its solution is a stepwise interpolating function f determined by n points $\theta = [\theta_1, ..., \theta_n]$ in monotonically decreasing order. It is solved by formulating the search of f as a quadratic optimization problem. However, monotonic regression generally suffers from the discontinuity of the stepwise function. Ref[10] proposes a modification of the quadratic optimization problem by penalizing the difference between adjacent fitted response values,$\theta_i$and θ_{i+1} (i in $[1, n-1]$), by using an L2 regularization term. The regression problem can be formulated for the monotonic and smooth monotonic case such as:

$$\begin{aligned} \min_{\theta} \sum_{i=1}^{n-1} (f(x_i, \theta) - y_i)^2 &= \\ & \min_{\theta} \sum_{i=1}^{n-1} (\theta_i - y_i)^2 \ \ (Monotonic) \\ & \min_{\theta} \sum_{i=1}^{n-1} (\theta_i - y_i)^2 + \sum_{i=1}^{n-1} \lambda_i (\theta_{i+1} - \theta_i)^2 \ \ (Smooth\ \ Monotonic) \\ s.t. \theta_i &\geq \theta_{i+1} \end{aligned} \tag{A.1}$$

where λ_i are selected as regularization parameters and y_i the data to model with the smooth monotonic function. The isotonic function is defined by adding additional constraints to the monotonic formula defined above such as:

$$\begin{aligned} \theta_1 &= 1 \ \ for f(0, \theta) = 1 \\ \theta_{i+1} - 2\theta_i + \theta_{i-1} &\geq 0 \ \ for\ \ Convexity\ \ f''(x_i) \geq 0 \end{aligned}$$

The Degradation-Correction Algorithm

We display here the methodology to correct the observations a and b displayed in Eq. 2. We also interpolate the measurements on channel B to get the same observing rate as in channel A equal to n. This algorithm is based on only one measurement to correct the two channels. A similar algorithm involving both channels is shown in ref.[11,12]. In practice, the choice of the correction algorithm does not influence significantly the correction-degraded TSI time series.

To start, at each iteration the ratio is computed between the initial signal a and corrected b (b_c). The ratio is an approximation of the degradation function which is modelled here with the smooth monotonic function discussed previously. Let us call the observations on both channels before starting the iterations $a_0(t) = a(t)$ and $b_0(t) = b(t)$. At the p-th iteration, the ratio is:

$$r_p(e_a(t)) = \frac{a_0(t)}{b_{p-1}(t)} \tag{A.2}$$

If we fit the ratio with one of the function described in Eq. 3, then $f_p(e_a(t)) = r_p(e_a(t))$, and the corrected observations are at the p-th iteration:

$$\begin{aligned} a_p(t) &= \frac{a_0(t)}{f_p(e_a(t))} \\ b_p(t) &= \frac{b_0(t)}{f_p(e_b(t))} \end{aligned} \tag{A.3}$$

The degradation function is then updated iteratively such as $d_c(.) = f_p(.)$. Here, we assume that the algorithm converges to the true signals (i.e. $a_c(.) \longrightarrow s(.)$, $b_c(.) \longrightarrow s(.)$, $d_c(.) \longrightarrow d(.)$). The convergence demonstration is left out as discussed in the section about the algorithm and in ref[12]. The pseudo-code of the algorithm can be written as follow.

$a_c \longleftarrow a_0$; $b_c \longleftarrow b_0$ (Initial estimate of corrected signals before iterations)

while not converged **do** ($||a_{p+1} - a_p||_2 / ||a_p||_2 + ||b_{p+1} b_p||_2 / ||b_p||_2 > \gamma$)

$r \longleftarrow \frac{a_c}{b_c}$ (Divide signals a_0 and b_c pointwise, i.e. $r[k] = a_0[k]/b_c[k]\ \forall k \in [n]$)

$f(.) \longleftarrow$ Fit Curves To $((e_a[k])\ k \in [n]\ ,\ (r[k]) k \in [n])$ (Learn mapping $f : e_a \longrightarrow f(e_a)$)

$a_c \longleftarrow \frac{a_0}{f(e_a)}$ (Correction update of signal a)

6 $b_c \longleftarrow \frac{b_0}{f(e_b)}$ (Correction update of signal b)

7 **End while**

8 $d_c(.) \longleftarrow f(.)$ (Final Estimate of Degradation Function)

9 **Return** $a_c[k]$, $b_c[k]$, $d_c(.)$ (Corrected signals and degradation function)

where $||.||_2$ is the Euclidean norm. γ is a threshold relatively small.

Approximation of the Log-likelihood in the Data Fusion Algorithm

We discussed in the section about the algorithm that the VIRGO/SOHO TSI data sets are large due to the high-recording rate. We then need to approximate the exact Gaussian Processes (GPs) by utilizing Sparse Gaussian Processes (SPGs), yielding a maximization problem of the lower bound of the log-likelihood $\log p(\mathbf{y}|\mathbf{x})$, with $\mathbf{x}$ and $\mathbf{y}$ defined as the time vector $\mathbf{x} = [t_i, t_i], i = 1 \ldots n$ and $\mathbf{y} = [a_c(t_i), b_c(t_i)]$ the corresponding corrected observations. The maximization problem follows[13]:

$$\log p(\mathbf{y}|\mathbf{x}) \geq -\frac{1}{2}\mathbf{y}^T (Q_\alpha + \sigma^2 \mathbf{I})^{-1}\mathbf{y} - \frac{1}{2}\log|Q_\alpha + \sigma^2\mathbf{I}| - \frac{n}{2}\log(2\pi) - \frac{1}{2\sigma^2} tr(k_\alpha(\mathbf{x},\mathbf{x}) - Q_\alpha), \tag{A.4}$$

where $Q_\alpha = k_\alpha(\mathbf{x},\mathbf{u})k_\alpha(\mathbf{u},\mathbf{u})^{-1}.k_\alpha(\mathbf{u},\mathbf{x})$, $\mathbf{u}$ is a vector of inducing points (small set of observations), which are used to estimate the initial parameters in α. $\mathbf{I}$ is the identity matrix, with $\sigma^2\mathbf{I}$ the noise component of the covariance matrix formulated as $diag([\sigma_a^2, \sigma_b^2, \sigma_a^2, \sigma_b^2, \ldots])$. We then estimate the kernel k_α by maximizing the right-hand-side of Eq. (A.4) with respect to $\mathbf{u}$ and α. It is important to emphasize that the training of k_α with the inducing points is to learn about the stochastic properties of the data, which allows to take into account short-term correlations in the observations. Further mathematical simplifications to estimate the kernel are voluntarily left out for clarity, but readers can refer to Ref.[12].

Supplementary Figures

Figures A.1 and A.2 display the ratio of the raw PREMOS observations over the degradation corrected measurements as a function of their exposure time. Figure A.3 shows the raw VIRGO observations and after degradation correction. Figure A.4 shows a comparison between all the datasets and our new TSI composite time series.

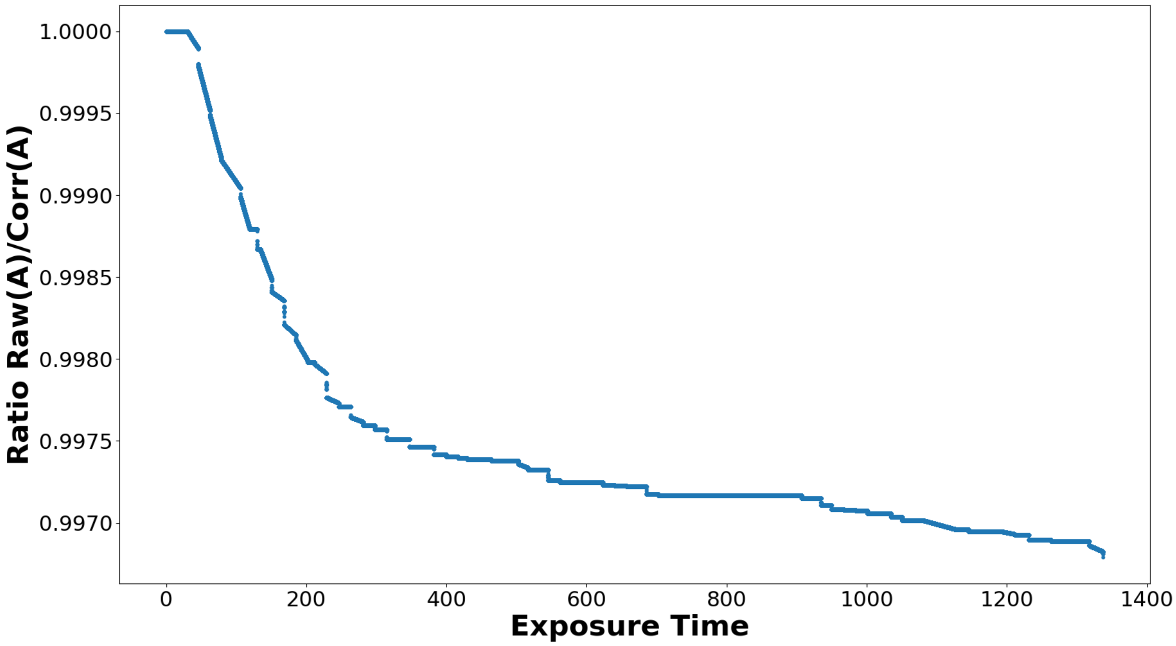

Figure A.1. Ratio of PREMOS/PMO6-PA Raw over corrected observations as a function of the exposure time.

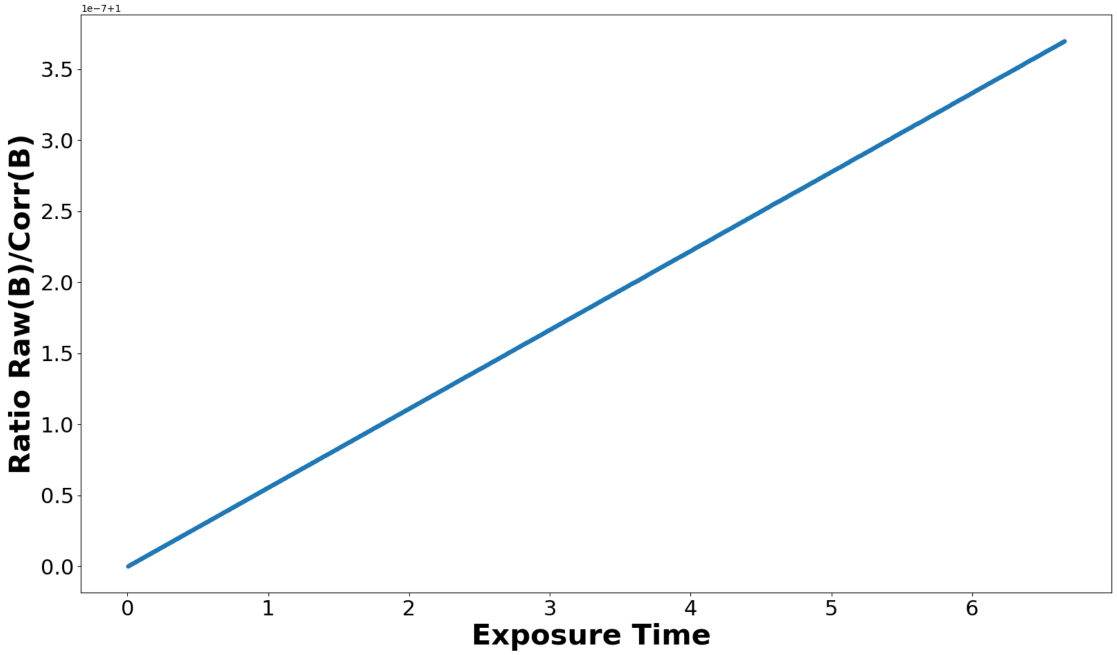

Figure A.2. Ratio of PREMOS/PMO6-PB Raw over corrected observations as a function of the exposure time.

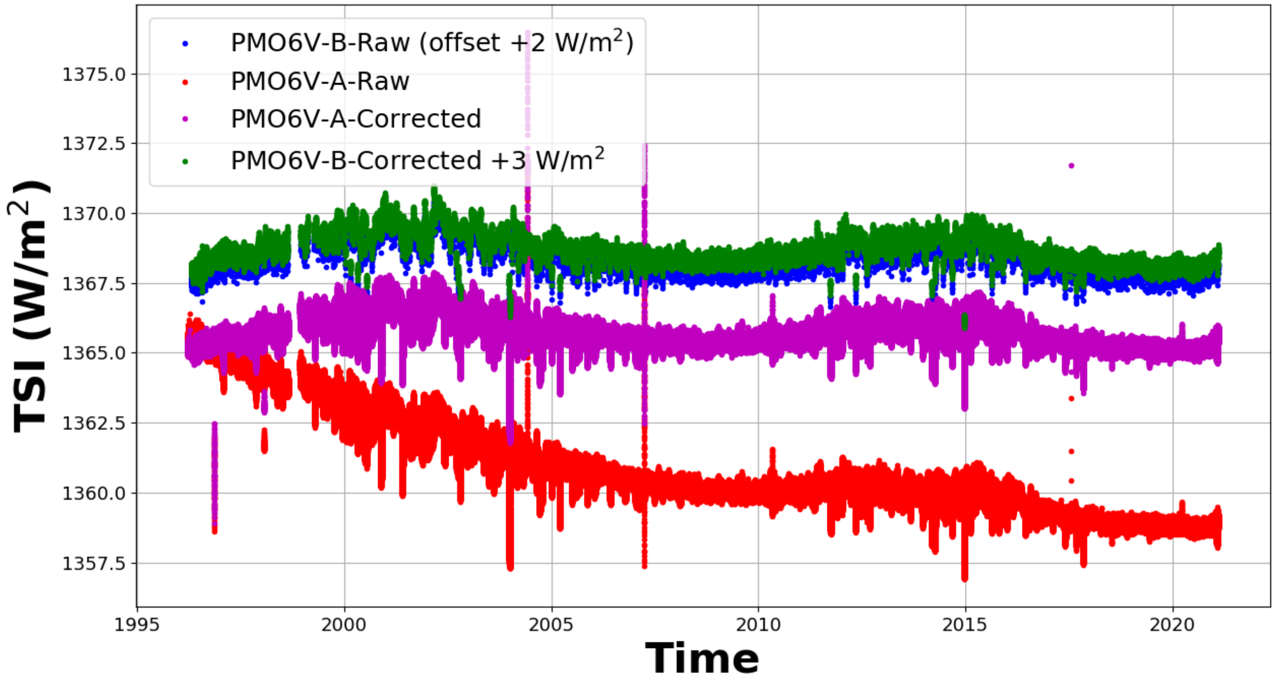

Figure A.3. VIRGO/PMO6V raw observations on both channel (VIRGO/PMO6V-A and VIRGO/PMO6V-B) before and after correction.

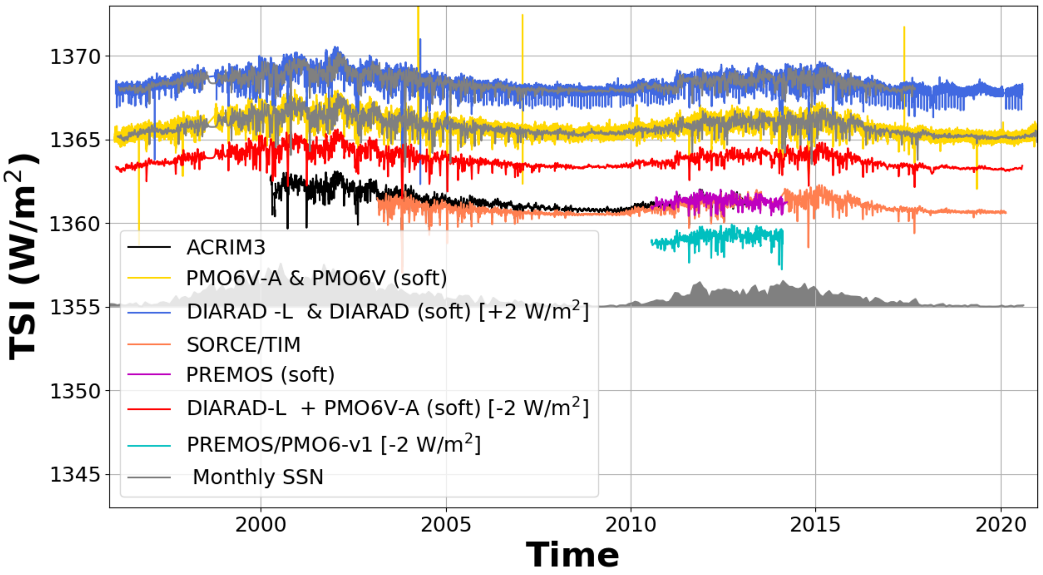

Figure A.4. Comparison of the various TSI composite time series used in this study with the degradation corrected dataset (PMO6V-A, DIARAD-L) (soft), the fused products (i.e. PMO6V-A with DIARAD -L, PREMOS) and the fused product for the main and back-up channel (grey) for both DIARAD and PMO6V. PREMOS (soft) is the resulting time series after fusing the degradation corrected PREMOS-PA and PREMOS-PB observations together with PREMOS v1. SSN is the time series of monthly averaged sunspot numbers.

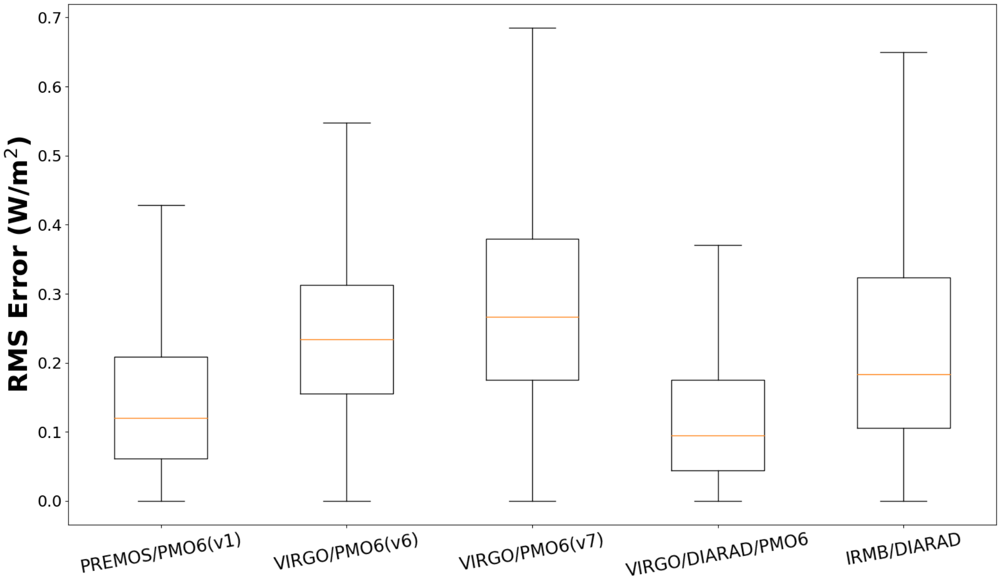

Figure A.5. Box-whiskers plot in terms of root mean square error (RMSE) difference between the previous releases and the new VIRGO TSI composite. The mean value is around 0.18 W/m^2, except for the comparison with VIRGO/DIARAD (soft) and PREMOS/PMO6 which is around 0.18 W/m^2 and 0.12 W/m^2. If we define 1 sigma interval as the inter quantile range (i.e. difference between the 25th and 75th percentile), we can establish that it is equal to ~ 0.24 W/m^2 (PMOD-v6), 0.27 W/m^2 (PMOD-v7), ~ 0.09 W/m^2 (IRMB/DIARAD), and ~ 0.12 W/m^2 for PREMOS-v1 time series. We thus decide to set 1 sigma interval (by averaging the three first values) equal to 0.2 W/m^2 for both VIRGO/PMO6 and VIRGO/DIARAD observations.In addition, the discrepancy shown with the large whiskers (up to 0.68 W/m^2 for PMOD-v7) is mainly due to spike differences. The fusion step of the algorithm smooths the time series, hence decreasing the amplitude of the large spikes.

References

1. Cessateur, G. *et al.* Solar irradiance observations with PREMOS filter radiometers on the PICARD mission: In-flight performance and data release. *Astron. Astrophys.* **588**, A126, DOI: 10.1051/0004-6361/201527577 (2016).
2. Schmutz, W. *et al.* The PREMOS/PICARD instrument calibration. *Metrologia* **46**, S202–S206, DOI: 10.1088/0026-1394/46/4/S13 (2009).
3. Fehlmann, A. *et al.* Fourth World Radiometric Reference to SI radiometric scale comparison and implications for on-orbit measurements of the total solar irradiacne. *Metrologia* **49**, DOI: 10.1088/0026-1394/49/2/S34 (2012).
4. Kopp, G., Lawrence, G. & Rottman, G. The Total Irradiance Monitor (TIM): Science Results. **230**, 129–139, DOI: 10.1007/s11207-005-7433-9 (2005).
5. Prša, A. *et al.* Nominal Values for Selected Solar and Planetary Quantities: IAU 2015 Resolution B3. *Astron. J.* **152**, 41, DOI: 10.3847/0004-6256/152/2/41 (2016). 1605.09788.
6. Fröhlich, C. *et al.* In-Flight Performance of the Virgo Solar Irradiance Instruments on Soho. *Sol. Phys.* **175**, 267–286, DOI: 10.1023/A:1004929108864 (1997).
7. Fröhlich, C. Solar Irradiance Variability Since 1978. Revision of the PMOD Composite during Solar Cycle 21. *Space Sci. Rev.* **125**, 53–65, DOI: 10.1007/s11214-006-9046-5 (2006).
8. Schmutz, W., Fehlmann, A., Finsterle, W., Kopp, G. & Thuillier, G. Total solar irradiance measurements with PREMOS/PICARD. In *American Institute of Physics Conference Series*, vol. 1531 of *American Institute of Physics Conference Series*, 624–627, DOI: 10.1063/1.4804847 (2013).
9. Ball, W. T., Schmutz, W., Fehlmann, A., Finsterle, W. & Walter, B. Assessing the beginning to end-of-mission sensitivity change of the PREcision MOnitor Sensor total solar irradiance radiometer (PREMOS/PICARD). *J. Space Weather. Space Clim.* **6**, A32, DOI: 10.1051/swsc/2016026 (2016).
10. Sysoev, O. & Burdakov, O. A smoothed monotonic regression via L2 regularization. Knowledge and Information Systems. *Knowl. Inf. Syst.* **59**, 197–218, DOI: 10.1007/s10115-018-1201-2 (2019).
11. Kolar, L., Šikonja, R. & Treven, L. Iterative Correction of Sensor Degradation and a Bayesian Multi-Sensor Data Fusion Method applied to TSI Data from Pmo6-V Radiometers - [available at $https://github.com/roksikonja/dslab_{v}irgo_{t}si$] (2019).
12. Kolar, L., Šikonja, R. & Treven, L. Iterative Correction of Sensor Degradation and a Bayesian Multi-Sensor Data Fusion Method. *ArXiv* DOI: arXiv:2009.03091 (2020).
13. Bauer, M., van der Wilk, M. & E., R. C. Understanding Probabilistic Sparse Gaussian Process Approximations. In *NIPS*, vol. 2016, DOI: arXiv:1606.04820 (2016).
